# Lymph node-derived donor encephalitogenic CD4^+ ^T cells in C57BL/6 mice adoptive transfer experimental autoimmune encephalomyelitis highly express GM-CSF and T-bet

**DOI:** 10.1186/1742-2094-8-73

**Published:** 2011-06-24

**Authors:** Petra D Cravens, Rehana Z Hussain, Tresa E Zacharias, Li-Hong Ben, Emily Herndon, Ramya Vinnakota, Doris Lambracht-Washington, Stefan Nessler, Scott S Zamvil, Todd N Eagar, Olaf Stüve

**Affiliations:** 1 Department of Neurology, University of Texas Southwestern Medical Center at Dallas, TX, USA; 2 Department of Pathology, University of Texas Southwestern Medical Center at Dallas, TX, USA; 3 Department of Neurology, University of Göttingen, Germany; 4 Department of Neurology, University of California, San Francisco, CA, USA; 5 Neurology Section, VA North Texas Health Care System, Medical Service, Dallas, TX, USA

## Abstract

Experimental autoimmune encephalomyelitis (EAE) is a relevant animal model for the human demyelinating inflammatory disorder of the central nervous system (CNS), multiple sclerosis (MS). Induction of EAE by adoptive transfer allows studying the role of the donor T lymphocyte in disease pathogenesis. It has been challenging to reliably induce adoptive transfer EAE in C57BL/6 (H-2_b_) mice. The goal of this study was to develop a reproducible and high yield protocol for adoptive transfer EAE in C57BL/6 mice. A step-wise experimental approach permitted us to develop a protocol that resulted in a consistent relatively high disease incidence of ~70% in recipient mice. Donor mice were immunized with myelin oligodendrocyte glycoprotein (MOG)_p35-55 _in complete Freund's adjuvant (CFA) followed by pertussis toxin (PT). Only lymph node cells (LNC) isolated at day 12 post immunization, and restimulated *in vitro *for 72 hours with 10 μg/mL of MOG_p35-55 _and 0.5 ng/mL of interleukin-12 (IL-12) were able to transfer disease. The ability of LNC to transfer disease was associated with the presence of inflammatory infiltrates in the CNS at day 12. Interferon gamma (IFNγ) was produced at comparable levels in cell cultures prepared from mice at both day 6 and day 12 post immunization. By contrast, there was a trend towards a negative association between IL-17 and disease susceptibility in our EAE model. The amount of GM-CSF secreted was significantly increased in the culture supernatants from cells collected at day 12 post immunization versus those collected at day 6 post-immunization. Activated CD4^+ ^T cells present in the day 12 LNC cultures maintained expression of the transcription factor T-bet, which has been shown to regulate the expression of the IL-23 receptor. Also, there was an increased prevalence of MOG_p35-55_-specific CD4^+ ^T cells in day 12 LNC after *in vitro *re-stimulation. In summary, encephalitogenic LNC that adoptively transfer EAE in C57BL/6 mice were not characterized by a single biomarker in our study, but by a composite of inflammatory markers. Our data further suggest that GM-CSF expression by CD4^+ ^T cells regulated by IL-23 contributes to their encephalitogenicity in our EAE model.

## 1. Introduction

Multiple sclerosis (MS) is an inflammatory-mediated demyelinating disease of the human central nervous system (CNS) that afflicts approximately 2.5 million individuals worldwide [1]. Experimental autoimmune encephalomyelitis (EAE) is the most commonly used animal model for studying the underlying disease mechanisms of MS and for testing potential new therapies [2-4]. Two methods are commonly used to induce EAE in rodents: (1) Active immunization and (2) adoptive transfer [2,4]. In active immunization, the experimental animal is inoculated with the encephalitogenic antigen in Complete Freund's Adjuvant (CFA) or other adjuvants. In addition, the administration of pertussis toxin (PT) is required for disease induction in many mouse strains. Following immunization with a dominant determinant, numerous immune competent cells undergo activation, including antigen presenting cells (APC) and effector cells.

The adoptive transfer model was developed to specifically test the role of antigen specific T cells in EAE pathogenesis [5-7]. For this purpose, donor mice are immunized with antigen in CFA, and their lymph nodes (LNs) or spleens are harvested 6-14 days later. Single cell suspensions are prepared from the harvested organs, and are re-stimulated *in vitro *with antigen and cytokines to select for a population of antigen-specific encephalitogenic T cells. After 3-8 days of culture, the cells are injected into recipient mice. In susceptible mouse strains, disease onset and disease severity can parallel that of EAE following active immunization.

While EAE induction by adoptive transfer is reliable and reproducible in several mouse strains [8-10], adoptive transfer in the C57BL/6 model (H-2_b_) has been problematic in our hands and in the hands of other investigators (personal communications). Specifically, disease incidence, time of disease onset, and disease severity after transfer of myelin oligodendrocyte glycoprotein peptide (MOG_p_)_35-55_-specific T cells are highly variable. Pre-conditioning of recipients with sub-lethal irradiation can be an additional confounding factor in the pathogenesis of CNS demyelinating disease. Overall, the C57BL/6 mouse is considered relatively resistant to EAE induction by adoptive transfer.

The C57BL/6 mouse is of particular interest to neuroimmunologists and neuroscientists for numerous reasons: (1) It is now the most commonly used mouse strain in actively induced EAE experiments; (2) most genetically-modified mouse strains are on the C57BL/6 background; (3) C57BL/6 mice are commonly backcrossed with transgenic mice or gene-deficient mice on the C57BL/6 background. Therefore, successfully inducing EAE in the C57BL/6 mouse via adoptive transfer is critical for studying the contribution of encephalitogenic T cells in clinical and paraclinical CNS autoimmune disease.

In this project, we identified and characterized a reliable and reproducible method of adoptively transferring EAE into immunocompetent C57BL/6 mice.

## 2. Materials and methods

### 2.1 Immunizing donor mice

C57BL/6 donor mice were purchased from (The Jackson Laboratories, Bar Harbor, MN) and bred at the University of Texas Southwestern Medical Center (UTSW) under specific pathogen free conditions. All animal protocols were approved by the UTSW Institutional Animal Care and Use Committee (IACUC). Donor mice were anesthetized with tribromoethanol (^®^Avertin; Sigma Aldrich, St. Louis, MO) 250 mg/kg intraperitoneally (i.p.) and then immunized subcutaneously (s.c.) with a homogenized emulsion of 200 μg/100 μL MOG_p35-55 _(C.S. Bio, Menlo Park, CA)/Complete Freund's Adjuvant (CFA)(DIFCO Laboratories, Detroit, Michigan, USA) supplemented with 2 mg/ml of mycobacterium tuberculosis H37RA (MT) (DIFCO Laboratories). 25 μl of emulsion was injected into the bilateral scapular and inguinal areas. In some experiments, the donor mice or the recipient mice were also injected i.p. with 200 ng/200 μL pertussis toxin (PT) (List Biological Laboratories Inc)/phosphate buffered saline (PBS) (Sigma Aldrich, St. Louis, MO) on the day of immunization and 2 days post immunization.

### 2.2 Preparing single cell suspensions

**Figure 1:**
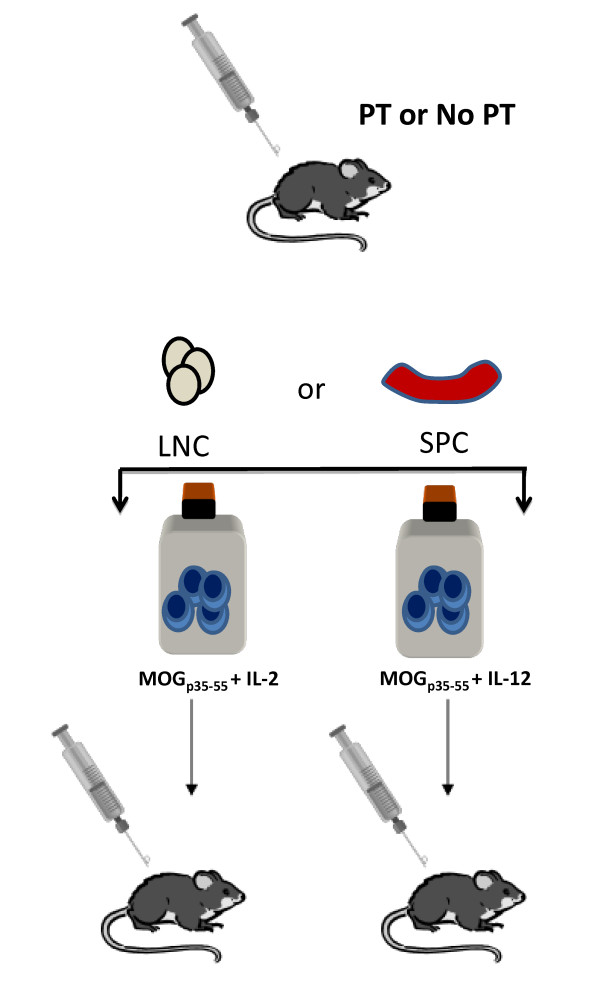
**General schematic for induction of EAE by adoptive transfer into C57BL/6 mice**. Groups of donor mice were immunized with CFA/(MOG)_35-55_. Some mice also received pertussis toxin (PT) on the day of immunization (day 0) and on day 2 post immunization. Lymph node cells (LNC) and splenocytes SPC) were harvested at either day 6 or day 12 post immunization and were restimulated *in vitro *with a combination of MOG_p35-55 _and IL-2, or MOG_p35-55 _and IL-12 for 3-8 days in tissue culture flasks. After specified days in culture, 10-20 × 10^6 ^cells in 200 μL PBS were injected into recipient mice. Mice were observed every day after injection for disease onset and clinically scored.

Lymph nodes cells (LNC) and splenocytes (SPC) were harvested from donor mice at 6 or 12 days post immunization (Figure 1). Organs were pressed through a 70 μm mesh into cold PBS. Cells were pelleted at 450*g *for 5 minutes at 4°C. SPC and LNC were resuspended in red blood cell lysing buffer (Sigma, St. Louis, MO) for 5 minutes. After three washes in with cold PBS, cells were counted with the hemocytometer. For counting, cells were diluted with 0.4% Trypan Blue (Sigma Aldrich, St. Louis, MO) to permit discrimination of dead cells.

### 2.3 Preparation of donor cells for adoptive transfer

LNC and SPC were cultured for 3-7 days in 75 ml tissue culture flasks (Corning, Corning, NY) at 7.5 million cells/mL in RPMI 1640 supplemented with 10% fetal bovine serum (FBS), 1.25% Hepes buffer,1% sodium pyruvate, 1% penicillin-strepyomycin, 1% glutamine, 1% non essential amino acids, 0.01% 0.05m-2 mercaptoethanol (2-ME) (Sigma Aldrich, St. Louis, MO). Cells were cultured with either 10 or 20 μg/mL of MOG_p35-55_. Also, cells were cultured with one of two cytokines: Interleukin-2 (IL-2) (R&D Systems, Minneapolis, MN) at 10 μg/mL or interleukin-12 (IL-12) (R&D Systems, Minneapolis, MN) at 0.5 ng/mL. For some experiments, cells were cultured in 24 well plates (Corning, Corning, NY) at 1 × 10^6 ^- 2 × 10^6 ^cells/well at 1 × 10^6 ^cells/mL in complete RPMI. Before adoptive transfer into recipient mice, cell viability was assessed by trypan blue exclusion.

### 2.4 Histology

Brains and spinal cords from naïve and immunized mice were collected into 10% formalin. Tissues were embedded in paraffin and sections were stained with H&E to detect immune cell infiltration, PAS to determine the presence of myelin breakdown products, and with Luxol Fast Blue to examine demyelination as described [11,12]. Tissues were scored by a blinded examiner.

### 2.5 Induction of EAE by Adoptive transfer

After 3-7 days of culture, the LNC and SPC were washed twice with room temperature PBS and 1 × 10^7 ^- 2 × 10^7 ^cells/200 μL PBS were injected i.p. into recipient male C57BL/6 mice. The mice were monitored daily for onset of EAE.

### 2.6 Clinical scoring

EAE was evaluated and scored according to following criteria: 0-no clinical abnormality; 0.5-limp tail only; 1-limp tail with mild hindlimb weakness; 2-limp tail with moderate hindlimb weakness; 3-hindleg paresis with or without mild forelimb weakness; 4-hindleg paresis with or without moderate forelimb weakness; 5-quadriplegia; and 6-dead or moribund [11-16].

### 2.7 Cytokine Analysis

Cytokines present in the cell culture supernatants of LNC and SPC harvested on Day 6 and Day 12 post immunization with MOG/CFA and restimulated in vitro for 72 hours in the presence of MOG_p35-55 _and IL-12, were quantified using the Cytometric Bead Array (CBA) kit for IFN-γ, Il-17 and GM-CSF (BD Biosciences). Data were acquired on a LSRII instrument (BD Biosciences, San Jose, CA) and analyzed using the BD Flowcap data analysis software.

### 2.8 Flow cytometry

For intra-cellular cytokine staining, LNC and SPC were stimulated with PMA (50 ng/ml) and ionomycin (750 ng/ml) directly ex-vivo at day 6 and day 12 post-immunization. GolgiPlug (1 μl/ml) was added to each well. After 5 hours stimulation, cells were collected, washed, and resuspended in staining buffer (4% FCS and 0.1% sodium azide in PBS). Fc receptors were blocked with anti-CD16/32, and the cell surface was stained with the following mAb: Anti-CD3, anti-CD4 (Invitrogen), anti-CD8 (ebioscience, San Diego, CA), and antiCD62L and anti-CD44 (BD Biosciences) mAbs for 30 min at 4°C. After washing twice with staining buffer, cells were fixed and permeabilized using Cytofix/Cytoperm solution (BD Biosciences) for 20 min at 4°C. Intracellular cytokines were detected with anti-IFNγ mAb and anti-IL-17 diluted in PermWash solution for 30 min at 4°C. Cells were washed, resuspended in staining buffer, and fixed in 1% paraformaldehyde. Fifty thousand to 100,000 CD4^+ ^events were acquired on the FACSAria (BD Biosciences) using the Diva Software package. Data was analyzed using FlowJo software (Tree Star, Ashland, OR). CD3^+ ^CD4^+ ^CD44^+^T lymphocytes were examined for expression of IFNγ and IL-17.

Expression of T-bet by LNC and SPC that were to be adoptively transferred into recipient mice, was examined by flow cytometry as previously described (Gocke et al J. Immunol 2007). Briefly, after the cell surface was stained as described above, cells were washed and incubated with Fixation/Permabilization buffer (ebioscience) for 1 hour at 22°C. After washing twice in Permeabilization buffer (ebioscience) cells were stained for 60 minutes at 22°C with PE conjugated anti-T-bet (Santa Cruz Biotechnology, Santa Cruz, CA) or isotype control Ab diluted in Permeabilization buffer. Cells were washed twice in permeabilization buffer and at least 50,000 CD4^+ ^events were collected on a FACSAria (BD Biosciences, San Jose, CA). Data was analyzed as described above.

For MOG MHC class II tetramer staining, cultured LNC and SPC prepared for adoptive transfer were stained with fluorochrome labeled antibodies against murine CD3, CD4, and CD44 (BD Biosciences, San Jose, CA), and MOG_38-49 _MHC class II tetramer or control MHC class II tetramers containing hCLIP_103-117 _(both tetramers were generously provided by the NIH Tetramer Core Facility). After the cells were stained, samples were acquired on a FACSAria flow cytometer (BD Biosciences) and the data analyzed using Flowjo software (Treestar, Ashland, OR). Cells were gated for live cells and then CD3^+ ^CD4^+ ^CD44^+ ^T lymphocytes were examined for tetramer reactivity. Cells that were MOG tetramer-positive and CD44^+ ^were considered MOG-specific and thus recently activated.

### 2.9 Statistical analysis

Every experiment was repeated at least once. Error bars represent SEM. The means of two normally distributed samples were compared by Student's *t*-test. A minimum of 4 experimental animals was included in each paradigm. P-values < 0.05 were considered significant.

## 3. Results

### 3.1 LNC but not SPC isolated from C57BL/7 donors on day12 post immunization and with PT were able to transfer disease into C57BL/6 recipients after 72 hour in vitro restimulation with myelin antigen and IL-12

There were numerous combinations of biological factors that have been implicated in encephalitogenicity of T cells in other commonly used strains of mice [8-10,17]. To test these combinations in C57BL/6 mice, our experiments were set up as shown in the schematic diagram in Figure 1. Multiple experimental paradigm that yielded negative results are outline below but not shown in Figure 1. SPC of C57BL/6 donors that had been treated with PT or not, did not lead to disease induction in recipient mice regardless of the culture condition tested (data not shown). Similarly to SPC, LNC of mice that had been treated with PT or not, 6 days after inoculation with MOG_p35-55 _did not transfer disease. *In vitro *treatment of day 6 donor cells with IL-2 during the culture period prior to adoptive transfer did not result in EAE in any of the recipient animals. PT injections of the recipient had no effect on disease incidence (data not shown). In one set of experiments, we tested whether an antigen-specific or non-antigen-specific inflammatory challenge would increase the encephalitogenic potential of adoptively transferred cells under otherwise suboptimal conditions. Incomplete Freund's adjuvant (IFA)/MOG_p35-55_, or IFA/ovalbumin (OVA), or IFA/proteolipid protein (PLP)_178-191_, or lipopolysaccharide (LPS) were administered to mice on day 24 post adoptive transfer of LNC obtained from PT-treated donor mice 12 days post inoculation with MOG_p35-55_, and cultured for 72 hours in the presence of antigen and IL-2. None of the recipient mice developed clinical signs of EAE (data not shown).

**Figure 2:**
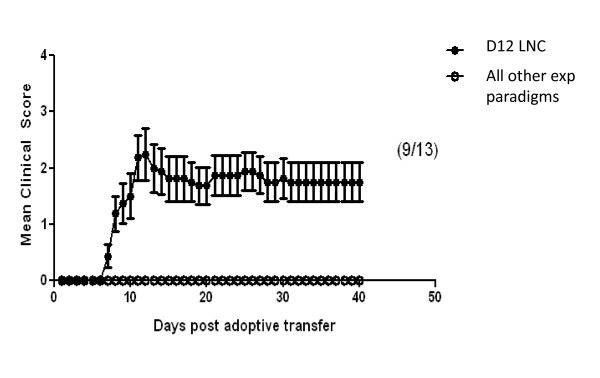
**EAE disease course in C57BL/6 mice after adoptive transfer of day 12 LNC**. The successful protocol required LNC that were harvested from donor mice that had been immunized 12 days previously and had been treated with PT. Following LNC isolation, the cells were restimulated *in vitro *with MOG_p35-55 _(10 μg/ml) and IL-12 (0.5 ng/ml) for 72 hours. Error bars represent ± SD. Clinical scores shown are from at least two independent experiments. The difference between the successful experimental paradigm (●)and all unsuccessful paradigms (O) with regard to disease activity was significant, starting on day 7 (p ≤ 0.001).

Of all of the combinations tested only one resulted in the ability to adoptively transfer EAE into C57BL/6 as shown in (Figure 2). The successful protocol required LNC that were harvested from donor mice that had been immunized 12 days previously and had been treated with PT. Following LNC isolation, the cells were restimulated *in vitro *with MOG_p35-55 _(10 μg/ml) and IL-12 (0.5 ng/ml) for 72 hours. At this timepoint, LNC but not SPC were able to induce EAE in recipient mice

**Figure 3:**
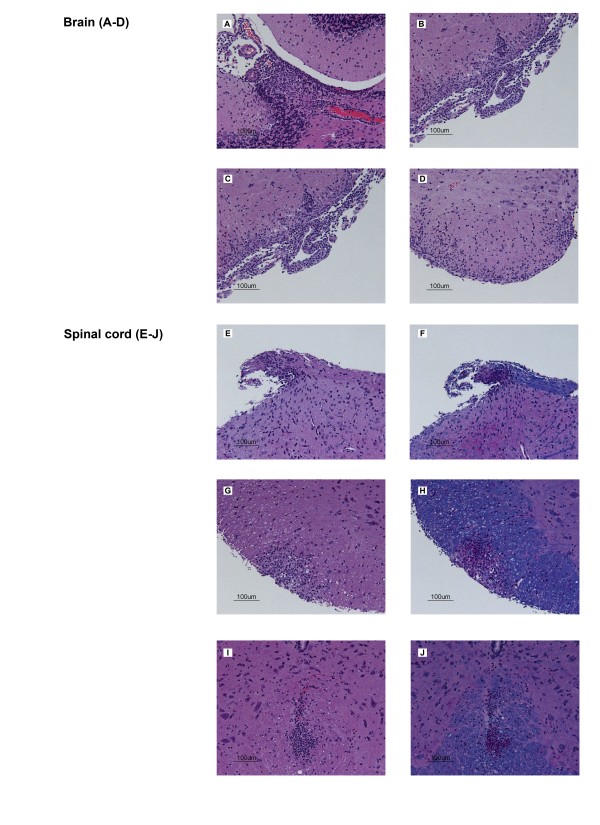
**Histopathological examination of the CNS of donor mice**. Representative data on the inflammatory infiltrates in the brain and spinal cord on day 12 are shown. **A**. Cerebellar cortex. **B**. Lateral brainstem. **C**. Posterior colliculus. **D**. Ventral brainstem. **E**. H&E and **F**. LFB/PAS/H&E of spinal cord dorsal root. **G**. H&E and **H**. LFB/PAS/H&E of lateral spinal cord white matter. **I**. H&E and **J**. LFB/PAS/H&E of ventral spinal cord white matter.

The CNS of donor mice was examined histologically to determine if the different immunization paradigms would result in an influx of leukocytes into the CNS, suggesting the generation of encephalitogenic CD4^+ ^T cells. Inflammatory infiltrates could be identified in sections of donor brain and spinal cord at day 12 but not in donor mice at day 6 post-immunization (Figure 3A, B, C, D, E, G, &3I), Table 1). Also at day 12 post immunization, but not on day 6 post immunization, there was PAS positive material throughout the CNS without detectable areas of demyelination (Figure 3 F, H, &3J).

**Table 1:** Presence of inflammatory infiltrates (+) or not (-) in the CNS of naïve and donor C57BL/6 mice at day 6 or day 12 post-immunization with CFA/MOG_p__35-5__5 _plus PT treatment

	Naive	Day 6	Day 12
Cerebellar cortex	-	-	+
Lateral brainstem	-	-	+
Posterior colliculus	-	-	+
Ventral brainstem	-	-	+
Spinal cord dorsal root	-	-	+
Lateral spinal cord white matter	-	-	+
Ventral spinal cord white matter	-	-	+

Tissues were processed for histopathological evaluation as described in the material and methods section.

### 3.2 Determining cytokine secretion by in vivo polarized T lymphocytes

**Figure 4:**
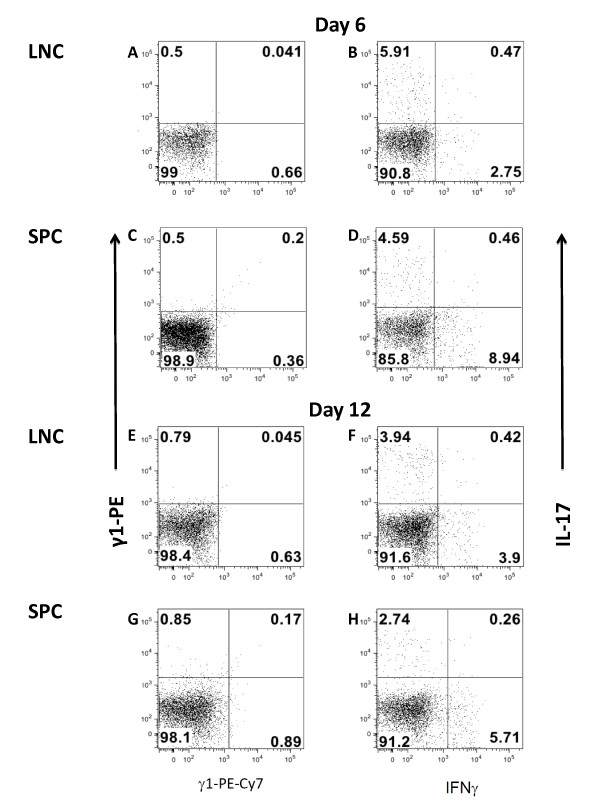
***Determining cytokine secretion by in vivo polarized T lymphocytes***. LNC and SPC from donor mice immunized 6 or 12 days previously were stimulated directly *ex-vivo *with PMA (50 ng/ml) and ionomycin (750 ng/ml) to determine the frequency of recently activated CD3^+^CD4^+^CD44^+ ^T cells present in these tissues prior to *in vitro *restimulation with antigen. IFNγ and Il-17 expressing T cells in LNC and SPC at day 6 and day 12 are shown in panels **B, D, F, and H**. Isotype controls are shown in panels **A, C, E, and G**. Data are representative of at least three independent experiments.

The successful induction of disease following the adoptive transfer of day 12 LNC as described above, and the detection of inflammatory CNS infiltrates in donor mice 12 days after immunization, we hypothesized that the production of proinflammatory cytokines is associated with the ability of the day 12 LNC, but not other cells, to transfer disease in C57BL/6 mice. LNC and SPC from donor mice immunized 6 or 12 days previously were stimulated directly *ex-vivo *to determine the frequency of recently activated CD3^+^CD4^+^CD44^+ ^T cells present in these tissues prior to *in vitro *restimulation with antigen. A comparable number of IFNγ and Il-17 expressing T cells were detected in both LNC and SPC at day 6 and day 12 (Figure 4 B, D, F, &4H).

### 3.3 Determining in vitro cytokine expression in LNC and SPC prior to adoptive transfer

**Figure 5:**
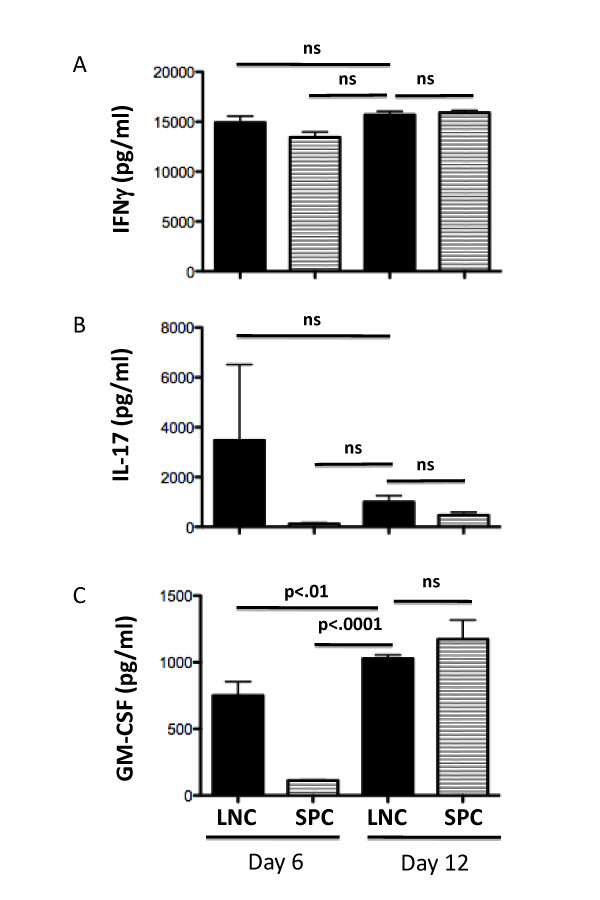
**Cytokine production *in vitro***. LNC and SPC harvested from donor mice at day 6 and day 12 post-immunization were cultured *in vitro *with 10 μg/ml MOG_35-55 _with 0.5 ng/ml IL-12. The concentration of IFN-γ (**A**), IL-17 (**B**), and GM-CSF (**C**) secreted by spleen and LNC after 72 hours in culture was determined by cytokine bead array (CBA). Data shown are representative of at least 2 independent experiments. ns = not significant.

To further define the cellular phenotype of donor cells prior to adoptive transfer in the paradigm that confers disease in C57BL/6 mice, LNC and SPC were restimulated on day 6 or day 12 *in vitro *for 72 hours in the presence of MOG_p35-55 _and IL-12. At this timepoint, cell culture supernatants were collected and the levels of IFNγ, IL-17 and GM-CSF were determined using cytokine bead arrays. IFNγ was produced at comparable levels in cell cultures prepared from mice at both day 6 and day 12 post immunization (Figure 5A). By contrast, there was a trend towards a negative association between IL-17 and disease susceptibility in our EAE model (Figure 5B). The amount of GM-CSF secreted was significantly increased in the culture supernatants of cells collected at day 12 post immunization versus those collected at day 6 post-immunization (Figure 5C).

### 3.4 Determining the levels of T-bet expression in cell populations to be adoptively transferred

**Figure 6:**
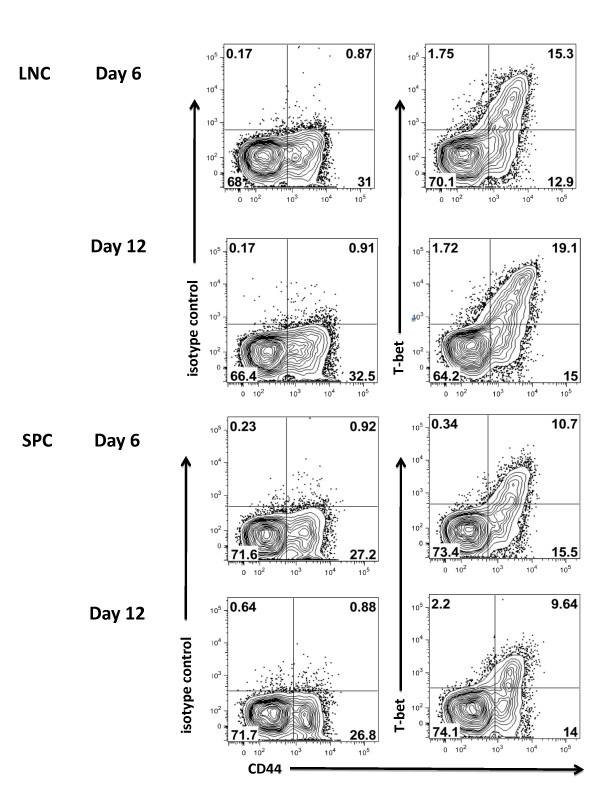
***Flow cytometry analysis of expression of T-bet by encephalitogenic CD4^+ ^T cells is maintained in Day 12 LNC***. C57BL/6 donor mice were immunized with CFA/MOG_p35-55 _followed by PT at day 0 and day2. At day 6 or day 12, LNC and SPC were harvested and cultured for 72 hours in the presence of MOG_p35-55 _and IL-12 and prepared for flow cytometry. CD3^+^CD4^+^CD44^+ ^T cells were examined for expression of T-bet. The percentage of T-bet^+ ^cell is indicated in the quadrants **B, D, F, & H**. Isotype controls are shown in panels **A, C, E, and G**. Data shown are representative of at least 3 independent experiments.

Expression of the transcription factor T-bet has been shown to play an important role in the development of encephalitogenic T cells [18-21]. To determine the encephalitogenic potential of the donor restimulated LNC and SPC to be adoptively transferred, we used flow cytometry to evaluate expression of T-bet. Cells from both day 6 and day 12 donor mice were restimulated *in vitro *for 72 hours with MOG_p35-55 _and IL-12. While we were able to detect CD44^+^T-bet^+ ^CD4^+ ^T cells in all culture conditions tested (Figure 6B, D, F, &6H), the frequency of CD44^+^T-bet^+ ^CD4^+ ^T cells was substantially higher in the day 12 LNC (Figure 6D), the only cells which were able to transfer disease into C57BL/6 mice.

### 3.5 Determining MOG-specificity in donor LNC and SPC

**Figure 7:**
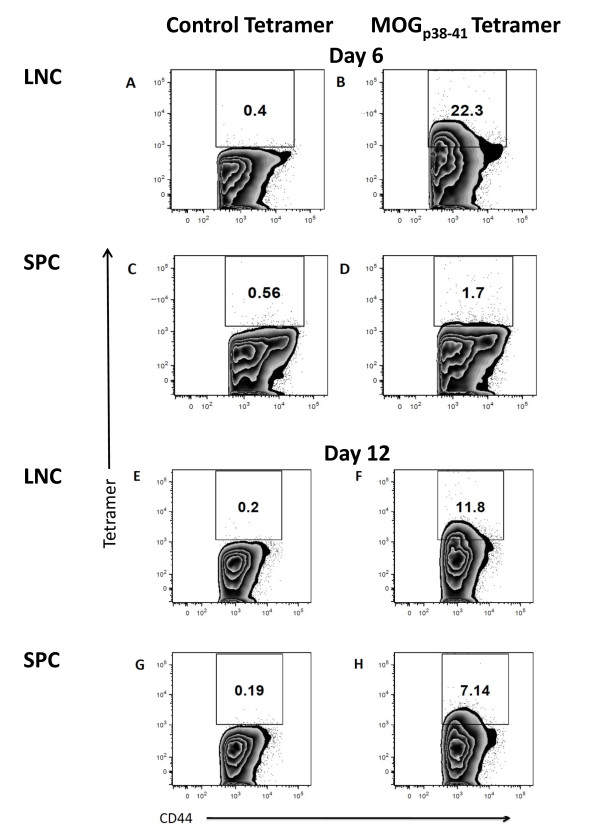
***Determining MOG-specificity in donor LNC and SPC***. At day 6 or day 12, LNC and SPC were harvested and cultured for 72 hours in the presence of MOG_p35-55 _and IL-12 and prepared for tetramer staining. CD3^+^CD4^+^CD44^+ ^T cells were examined for staining with MOG_38-49 _MHC class II tetramer, or control MHC class II tetramers containing hCLIP_103-117_. The percentage of MOG_38-49 _MHC class II tetramer positive cell is indicated in the regions **B, D, F, & H**. Control MHC class II tetramers containing hCLIP_103-117 _staining is shown in panels **A, C, E, and G**. Data shown are representative of at least 3 independent experiments.

In order to determine the frequency of MOG_p35-55_-specific T cells present within the population of donor cells to be adoptively transferred into recipient mice, MOG_38-49 _tetramer staining was performed on LNC and SPC collected from donor mice that had been immunized with MOG_p35-55_/CFA 6 days or 12 days previously. When CD3^+ ^CD4^+ ^CD44^+ ^T lymphocytes were stained after 72 hours of culture in the presence of MOG_p35-55 _and IL-12, a substantial number of MOG_p35-55_-specific CD4+ T cells were detected in day and day 12 LNC, and day 12 SPC (Figure 7 B, F, &7H).

## 4. Discussion

In a systematic, step-wise approach, we identified a paradigm that leads to reliable and reproducible induction of EAE by adoptive transfer in C57BL/6 mice, a mouse strain that is widely considered relatively disease resistant to this method of EAE induction. We made several interesting observations that led to the development of this experimental approach.

We observed that PT injections of the donor mice were required for successful adoptive transfer of EAE into C57BL/6 mice. PT treatment of recipient mice had no apparent effect on disease susceptibility. PT is predominantly used as an adjuvant for EAE disease induction by active immunization. Traditionally it was thought that PT disrupts the blood-brain barrier (BBB) by amplifying an inflammatory antigen-specific autoimmune response [22]. Other proposed mechanisms of PT in EAE disease induction include; the prevention of anergy, the induction of autoantigen-specific T cells [23], the abrogation of T-cell tolerance [24], and the irreversible inhibition of G proteins and other second messengers than may affect signaling pathways in the T cell or the APC that regulates T-cell differentiation [25]. In this regard, Shive and coworkers demonstrated that PT stimulates APCs resulting in the differentiation of T helper cells towards a Th1 phenotype [26]. More recently, Hofstetter and colleagues showed that co-injection with PT during active immunization activates APCs in the peripheral lymphoid organs and the CNS by inhibiting signaling through G proteins rather than signaling through Toll-like receptors [27]. In the adoptive transfer model of EAE, the transferred antigen-specific encephalitogenic T cell is less dependent on reactivation in CNS than in the active immunization model but interaction with DCs in the perivascular spaces and meninges is necessary for the entry of encephalitogenic T cells into the CNS parenchyma [28]. Our data suggest that PT treatment of the donor alone is sufficient to promote an encephalitogenic T cell phenotype that when transferred into healthy C57BL/6 recipients can result in the development of EAE.

With regard to cytokine expression by T cells to be adoptively transferred we focused on IFNγ, IL-17, and GM-CSF. In the past two decades, TH1 cells that express IFNγ, tumor necrosis factor beta (TNF)-ß, IL-2, and nitric oxide [29] have been implicated in EAE pathogenesis. IFNγ is considered the signature cytokine of TH1 cells, which activate myeloid cells to promote cell-mediated immunity. Perhaps the most convincing evidence to support a pathogenic role of IFNγ in CNS autoimmune disease was derived from an open-label, randomized clinical trial, in which 18 patients with MS received 1 μg, 30 μg, or 1000 μg of recombinant IFNγ i.v. twice a week for four weeks [30]. During treatment, 7 patients experienced an exacerbation. The trial investigators also detected an increase in circulating monocytes bearing major histocompatibility complex (MHC) II surface antigen, suggesting that clinical attacks during treatment were immunologically mediated. Interestingly, studies performed in IFNγ deficient mice or in mice treated with anti-IFNγ mAb developed more severe EAE [31-34]. Therefore the contribution of IFNγ to EAE pathogenesis remains unclear. We were able to detect both IFNγ in cell culture supernatants in all experimental paradigms, suggesting that TH1 cells were present in the adoptively transferred cells. Our data suggest that IFNγ expression by T cells is not sufficient for encephalitogenicity in this EAE model.

More recently, TH17 cells were identified as a distinct lineage of CD4^+ ^T helper cells that may facilitate the initiation and perpetuation of CNS autoimmune diseases [35]. TH17 cells synthesize IL-17, which mediates proinflammatory and allergic responses. The role of IL-17 as a causal factor in the etiology of EAE has remained somewhat controversial as mice deficient in IL-17 can develop EAE [32,36-40]. In our model of adoptive transfer and in the C57BL/6 mouse strain it appears that high expression of IL-17 by transferred donor cells is not absolutely required for disease induction. Furthermore, high expression of IL-17 by adoptively transferred day 6 LNC is not sufficient to induce CNS autoimmune disease. However, adoptive transfer EAE may be a multi-phasic disease. O'Connor et al recently showed in a C57BL/6 adoptive transfer model that purified antigen-specific TH1 cells are highly pathogenic, whereas transfer of purified TH17 cells does not cause disease [39]. Activated TH1 cells could readily access the non-inflamed CNS of recipient mice, whereas TH17 cells only appear in the CNS of mice with established EAE. These data and our own observations suggest that a high number of TH17 donor cells may not be required for disease induction, but may be critical to sustain CNS inflammation. TH17 cells may be recruited from the host during the amplification of the initial inflammatory response.

Other investigators recently showed that GM-CSF may play critical role in different models of active and passive EAE [41-43]. Our own results indicated that GM-CSF is secreted by LNC and SPC after antigen re-stimulation in the presence of IL-12. While GM-CSF was highly expressed by day 12 encephalitogenic LNC in our EAE model, it was also highly expressed by day 12 non-encephalitogenic SPC.

The transcription factor T-bet has recently been shown to be critical for encephalitogenicity [20,21]. Its precise role in EAE and MS pathogenesis remains to be fully elucidated. T-bet has pleiotropic biological effects, among one of which is the regulation of expression of the IL-23 receptor [21,44]. In turn, IL-23 was recently shown to regulate the expression of GM-CSF by CD4^+ ^Tcells [42]. When we analyzed both splenocytes and LNC for expression of T-bet immediately before transfer into recipient mice, we found that T-bet^+ ^cells were maintained in the day 12 LNC, the only cell population able to transfer EAE. While T-bet^+ ^cells were present in the CD4^+ ^populations of the day 6 splenocytes and LNC, as well as day 12 splenocytes, these cells failed to transfer EAE.

In summary, we have identified an algorithm that leads to a reproducible induction of adoptive transfer EAE in C57BL/6 mice. There was no single biomarker associated with donor T cell encephalitogenicity. Instead, adoptively transferred day 12 LNC have the potential of reproducibly inducing EAE in the C57BL/6 mice are characterized by a composite of inflammatory markers that include the expression of GM-CSF and T bet. Our data further suggest that GM-CSF expression by CD4^+ ^T cells regulated by IL-23 contributes to their encephalitogenicity in our EAE model. Finally, there was an increased prevalence of MOG_p35-55_-specific CD4^+ ^T cells in day 12 LNC after *in vitro *re-stimulation. Our protocol is currently being verified in genetically-modified mice on the C57BL/6 background.

## Competing interests

The authors declare that they have no competing interests.

## Authors' contributions

PC, RH, TZ, and LB performed all the experiments. EH performed the histological evaluation of tissues. DLW, RV, SN and TE assisted with data analysis. PC, RH and OS designed the study, co-ordinated the experiments, prepared the figures and wrote the manuscript. TE and SZ contributed to the manuscript. All authors read and approved the final manuscript.
